# The senile plaque: Morphological differences in APP knock‐in mice brains by fixatives

**DOI:** 10.1002/brb3.2953

**Published:** 2023-03-06

**Authors:** Rikuya Yoshimura, Tomohiro Miyasaka, Satoru Funamoto, Takashi Saito, Takaomi C. Saido, Masaya Ikegawa, Nobuto Kakuda

**Affiliations:** ^1^ Faculty of Life and Medical Sciences Doshisha University Kyoto Japan; ^2^ Center for Research in Neurogenerative Diseases Doshisha University Kyoto Japan; ^3^ Laboratory for Proteolytic Neuroscience RIKEN Brain Science Institute Saitama Japan; ^4^ Department of Neurocognitive Science, Institute of Brain Science Nagoya City University Graduate School of Medicine Sciences Aichi Japan

**Keywords:** Amyloid β protein, Senile plaque

## Abstract

The morphology of senile plaques depends on the APP knock‐in mice brain fixative. Solid forms of senile plaques were detected in APP knock‐in mice after formic acid treatment with Davidson's and Bouin's fluid fixative as the brain of AD patients. Aβ42 was deposited as cored plaques and Aβ38 accumulated around Aβ42.

## Graphical abstract

This article showed suitable fixatives need to detect the form of senile plaque and its associated proteins in Alzheimer's disease model mice brains.

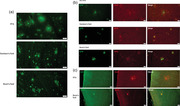

## 1 METHODS

### 1.1 Reagents

Anti‐Aβ N‐terminal‐specific antibody (82E1) and anti‐Aβ42 (44A3) monoclonal antibodies were purchased from IBL (Gunma, Japan). The anti‐Aβ38 monoclonal antibody (6A1) was made in the laboratory. Alexa Fluor 488 secondary antibody and Alexa Fluor 555 conjugate streptavidin were purchased from Thermo Fisher (MA, USA). For mouse brain tissue fixation and deparaffinization, paraformaldehyde, ethanol, acetate, 2,4,6‐trinitrophenol, and xylene were purchased from Fujifilm‐Wako (Osaka, Japan).

### 1.2 Immunohistochemistry

Four‐month, 10‐month of age APP *^NL‐G‐F/NL‐G‐F^*, and 12‐month of age APP *^NL‐F/NL‐F^* knock‐in mice brain tissues were subjected to 4% PFA in phosphate buffered‐saline (PBS), Davidson's fluid, or Bouin's fluid fixative after PBS perfusion for 1 day. The brains were sequentially dehydrated with 70%, 80%, 90%, and 3 times 100% ethanol and 3 times xylene (Fiji film‐Wako, Osaka, Japan) for 1 h each. Brain tissues were embedded in paraffin and sliced at 6 μm thickness. Deparaffinized sections were dipped in 98% formic acid for 4 min to enhance the staining for Aβs. Epitope retrieval was performed with autoclave treatment in 10 mM citrate buffer (pH 6.0) at 110°C for 10 min. These sections were blocked with 10% normal goat serum in PBS. Primary antibodies against Aβ (1 μg/mL) were incubated with the sections at 4°C overnight. In the case of merging Aβ38 and Aβ42, a biotinylated Aβ42 antibody was used. Alexa Fluor 488 conjugated secondary antibody and Alexa Fluor 555 streptavidin conjugate were used to visualize Aβ.

### 1.3 Immunofluorescence microscopy

Immunohistochemically stained APP knock‐in mouse brain sections with each antibody were analyzed with an Olympus BX50 (Tokyo, Japan). BX50 was used for high‐magnification analysis. Filter sets were used U‐MNIBA (Olympus, Tokyo, Japan) for Aβ 38 and U‐MWIG (Olympus, Tokyo, Japan) for Aβ42 detection. In the case of the whole sectioning images of the APP*^NL‐G‐F/NL‐G‐F^* mouse brain, an Olympus APX100 (Tokyo, Japan) was used for analysis. A Filter set was used U‐FBNA (Olympus, Tokyo, Japan).

## 2 RESULTS AND DISCUSSION

**FIGURE 1:**
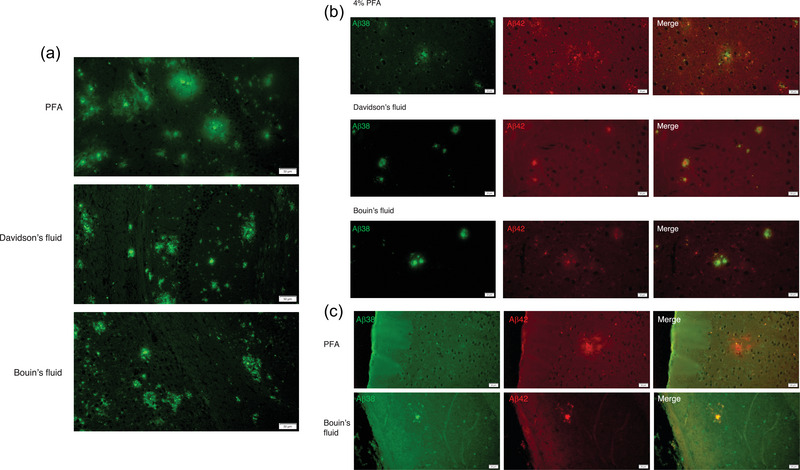
Different fixatives showed different SP morphology in the APP knock‐in mice brains. (A) Different SP morphologies are shown in the section with each fixative section (10‐month APP*^NL‐G‐F/NL‐G‐F^*). The SPs showed a solid form on the section of Davidson's fluid and Bouin's fluid compared with 4% PFA in the 10 months of APP *^NL‐G‐F/NL‐G‐F^* mouse brains. Scale bars indicate 50 μm. (B) Aβ38 showed diffuse‐like accumulation in the 4% PFA section. Aβ38 accumulated around Aβ42 with Bouin's fluid and Davidson's fluid of APP*^NL‐G‐F/NL‐G‐F^* mouse brains. Scale bars indicate 20 μm. (C) SP forms from 12‐month APP*^NL‐F/NL‐F^* brains compared to PFA and Bouin's fixatives. Scale bars indicate 20 μm.

Senile plaque (SP), one of the pathological hallmarks of Alzheimer's disease (AD) patient brains, can reproduce in its pathological model mouse brain. Amyloid β precursor protein (APP) *^NL‐G‐F/NL‐G‐F^* and APP *^NL‐F/NL‐F^* knock‐in mice are one of the most useful model mice in the global AD basic neurosciences and pharmacological sciences (Arroyo‐García et al., 2021; Blume et al., 2022; Hongo et al., 2020; Saito et al., 2014). To understand the specific morphology of SP in AD model mice brains, it is necessary to find SP‐associated molecules. Therefore, we focused on SP detection methods to assess the morphology of SP in these mice brains. The generated amyloid β (Aβ) in APP *^NL‐G‐F/NL‐G‐F^* has made SPs in the brain since the age of 2 months. This generated Aβ has an Arctic genetic mutation at the E22 amino acid position and is more prone to aggregate than wild‐type Aβ sequencing (Nilsberth et al., 2001). In previous studies, SPs in APP *^NL‐G‐F/NL‐G‐F^* and another AD mouse model were detected with antibodies after formic acid treatment or with thioflavin‐S (Emre et al., 2022; Reinert et al., 2016; Saito et al., 2014; Sasaguri et al., 2022). We followed this formic acid treatment to 10‐month‐old APP *^NL‐G‐F/NL‐G‐F^* brain sections. The antibody against the N‐terminus of Aβ, 82E1, showed diffuse‐like SPs around cored plaques, in which brain tissue was subjected to 4% paraformaldehyde (PFA) (Figure 1A). Thioflavin‐S detected the dense morphology of SP without formic acid treatment (Emre et al., 2022; Saito et al., 2014), but it cannot distinguish Aβ species from those of SP. To investigate the reason for this diffuse‐like SPs staining, 10‐month‐old APP *^NL‐G‐F/NL‐G‐F^* brain tissues were immediately subjected to Davidson's and Bouin's fluid fixative after saline perfusion. In these paraffin‐embedded sections, the antibody 82E1 showed undiffused SP forms in the section after formic acid treatment (Figure 1A). These undiffused SP morphologies were shown in the whole sections (Figure S1) Next, SPs stained with both anti‐Aβ38 and anti‐Aβ42 antibodies in 4% PFA, Davidson's fluid, and Bouin's fluid fixation sections in the 10‐month‐old APP *^NL‐G‐F/NL‐G‐F^* brain tissues. In the PFA fixed sections, Aβ38 showed diffuse‐like distribution as shown in 82E1 staining in Figure 1A, and Aβ42 accumulated in the core of SP (Figure 1B). However, in Davidson's fluid and Bouin's fluid fixed sections, Aβ38 accumulated around Aβ42 without halation, and Aβ42 accumulated in the core of SP (Figure 1B). To exclude the effect for the Arctic mutation, 12‐month of age APP *^NL‐F/NL‐F^* mice were used which carries wild‐type Aβ38 and Aβ42 sequencing. The left brain tissue was dipped in the 4% PFA fixative, right brain tissue was dipped in the Bouin's fixative from the same APP *^NL‐F/NL‐F^* after saline perfusion. The overlapped Aβ38 and Aβ42 were detected in the SP from Bouin's fixative (Figure 1C). However, Aβ38 could not detect from the PFA fixative. Only Aβ42 could be detected in the PFA section (Figure 1C). These results mean that PFA could fix Aβ42, although, Aβ38 could not be sufficiently fixed against formic acid treatment in wild‐type Aβ sequencing by PFA. To gain further insight into age dependency, 4‐month‐old APP *^NL‐G‐F/NL‐G‐F^* brains were stained after formic acid treatment. The number of SPs in the 4‐month‐old mice section was lower than that of those in the 10‐month‐old, but the staining patterns of Aβ42 were the same as those at 10 months of age in each fixative (Figure S2). The diffuse‐like morphology of Aβ38 did not depend on the age of the mice (Figure S2). These results indicate that Davidson's and Bouin's fluid fixative could be appropriate methods for the morphology of SP analysis rather than PFA fixative. Here, another epitope retrieval was performed with autoclave treatment in 10 mM citrate buffer in the PFA and Bouin's fixation section of 10‐month‐old APP *^NL‐G‐F/NL‐G‐F^*. This method also showed diffuse‐like Aβ38 in the PFA section. Importantly, Aβ42 was barely detectable in both PFA and Bouin's fixative (Figure S3). The autoclave treatment with citrate buffer might not have enough epitope retrieval effect for the Arctic mutant of Aβ42 in the APP *^NL‐G‐F/NL‐G‐F^* brains. Why did these discrepancies in SP morphology show in APP knock‐in mice brains with fixatives? The SPs bear in APP *^NL‐G‐F/NL‐G‐F^* brains within a few months. Thus, the physiological characterization of SP would still be softer and more flexible in these mice brains. Even though the brain was fixed with 4% PFA, formic acid will dissolve the SP in mice brain sections. On the other hand, Davidson's and Bouin's fluid fixative may be able to fix SP more strongly than PFA. Therefore, appropriate fixation and immunohistochemical methods are required to investigate SP morphology. These methods have the potential to accurately locate SP‐associated proteins in the brain of APP knock‐in mice and other AD model mice. Furthermore, as with previous human brain studies (Kakuda et al., 2017; Kakuda et al., 2020), it can compare the SPs of each Aβ species.

## AUTHOR CONTRIBUTIONS

R.Y. performed the experiments. T.M. technically supported immunohistochemistry. S.F. is responsible for APP knock‐in mice at Doshisha University. T.S. and T.C.S. generated APP knock‐in mice. M.I. supported the experiments. N.K. designed and performed the experiments and wrote the manuscript.

### ETHICS STATEMENT

The present study was approved by the animal ethics committees of Doshisha University (S.F.).

### PEER REVIEW

The peer review history for this article is available at https://publons.com/publon/10.1002/brb3.2953.

## Supporting information

Figure S1. Different SP morphologies are shown in the whole section with each fixative. Scale bars indicate 1 mm.
Figure S2. Comparison of the SP morphology with each fixative in the 4‐month age of APP *^NL‐G‐F/NL‐G‐F^* mouse brains. Aβ38 showed diffuse‐like morphology on the PFA fixative section, but the solid form of SP was shown in Davidson's and Bouin's fixative sections at 10 months age of APP *^NL‐G‐F/NL‐G‐F^* mouse brains.
Figure S3. Epitope retrieval with citrate buffer barely detects Aβ42. The section from 4% PFA and Bouin's fixative autoclaved with 10 mM citrate (pH 6.0). Aβ38 could detect both PFA and Bouin's fixative. A less Aβ42 was detected on the PFA section, but it could not be detected in Bouin's fixative. White arrows indicated the core of SP. Scale bars indicate 20 μm.

## Data Availability

All data and materials are included in this article and supplementary information files.
