# Supplementary material for: The senile plaque: Morphological differences in APP knock‐in mice brains by fixatives
Source: Brain Behav. 2023 Mar 6;13(4):e2953. doi: 10.1002/brb3.2953 (PMC10097081; doi:10.1002/brb3.2953)
Supplement: Supplementary file 1 — Figure S1. Different SP morphologies are shown in the whole section with each fixative. Scale bars indicate 1 mm. Figure S2. Comparison of the SP morphology with each fixative in the 4‐month age of APP NL‐G‐F/NL‐G‐F mouse brains. Aβ38 showed diffuse‐like morphology on the PFA fixative section, but the solid form of SP was shown in Davidson's and Bouin's fixative sections at 10 months age of APP NL‐G‐F/NL‐G‐F mouse brains. Figure S3. Epitope retrieval with citrate buffer barely detects Aβ42. The section from 4% PFA and Bouin's fixative autoclaved with 10 mM citrate (pH 6.0). Aβ38 could detect both PFA and Bouin's fixative. A less Aβ42 was detected on the PFA section, but it could not be detected in Bouin's fixative. White arrows indicated the core of SP. Scale bars indicate 20 μm. [file BRB3-13-e2953-s001.pptx]

## Slide 1
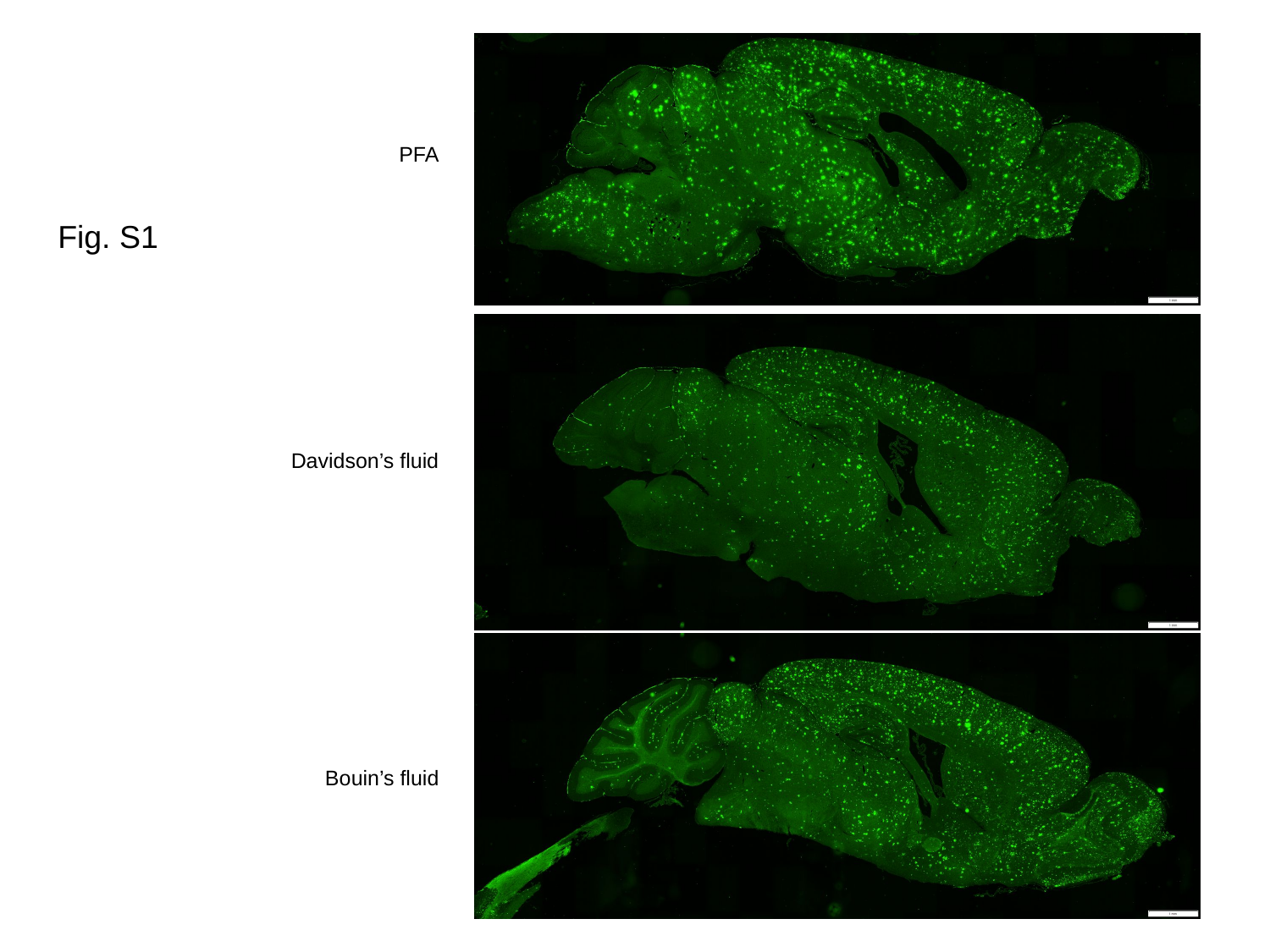

PFA
Fig. S1
Davidson’s fluid
Bouin’s fluid

## Slide 2
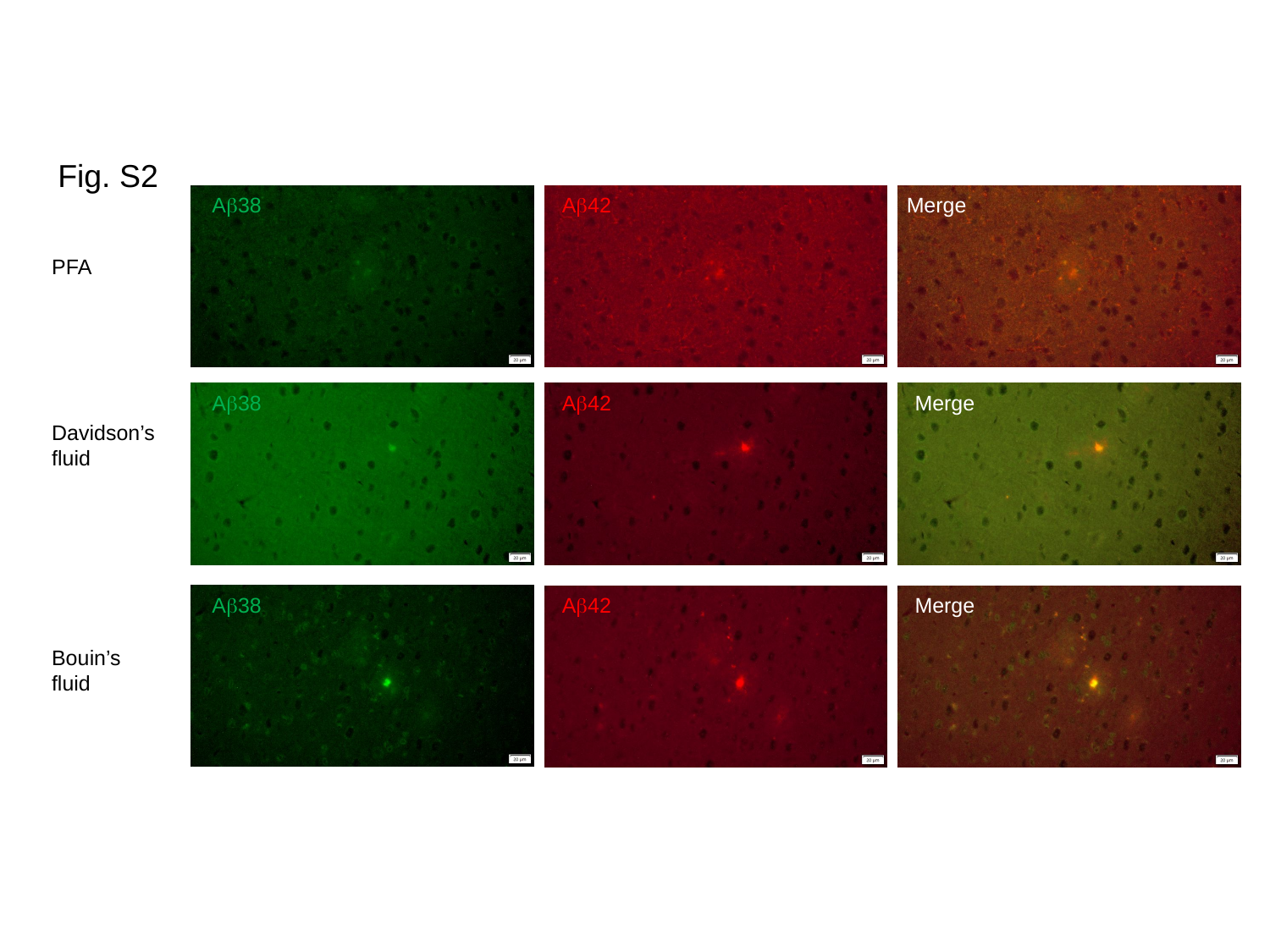

Fig. S2
Ab38
Ab42
Merge
PFA
Ab38
Ab42
Merge
Davidson’s
fluid
Ab38
Ab42
Merge
Bouin’s
fluid

## Slide 3
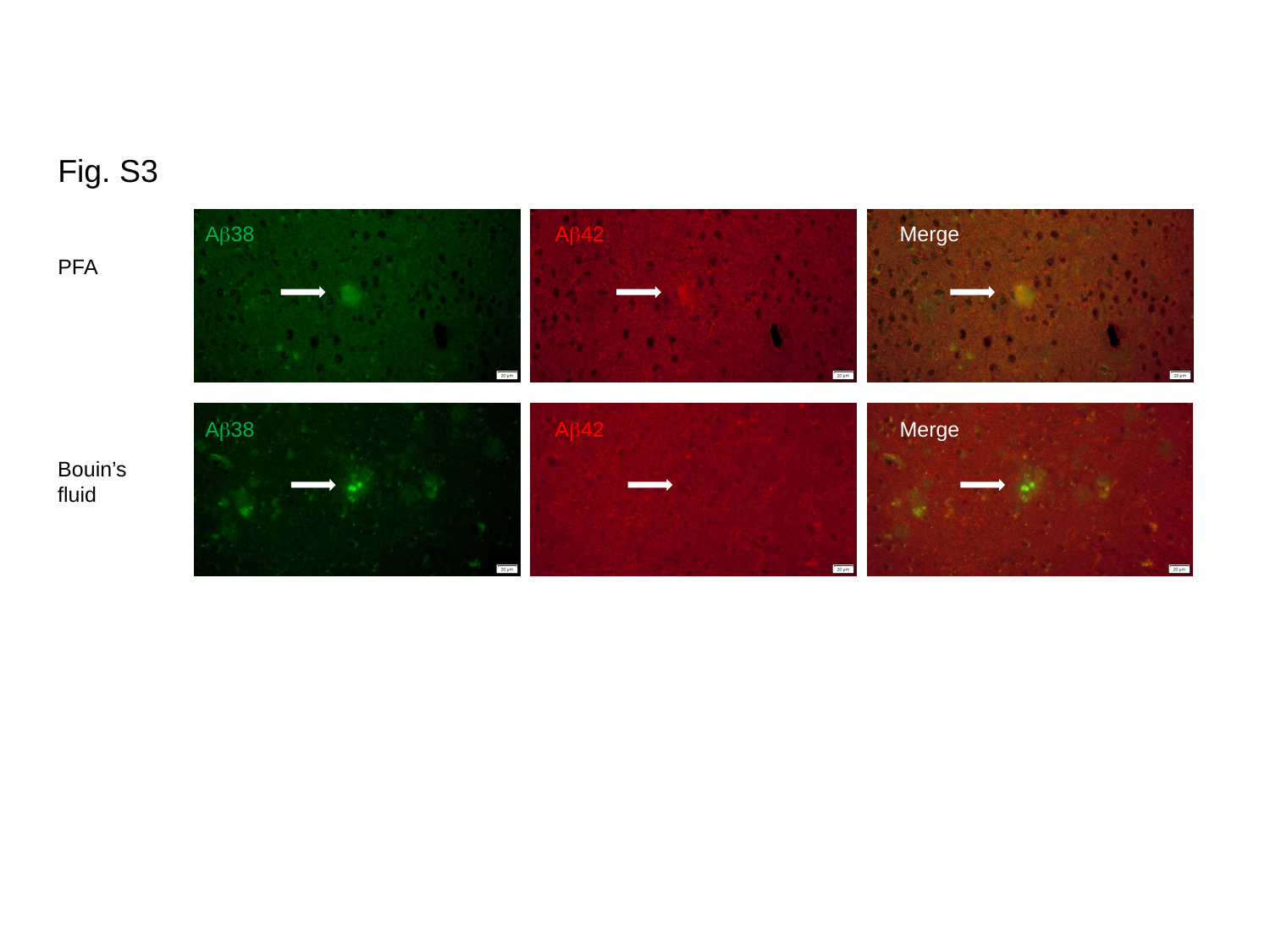

Fig. S3
Ab38
Ab42
Merge
PFA
Ab38
Ab42
Merge
Bouin’s
fluid
